# Compilation and Analysis of Web-Based Orthopedic Personalized Predictive Tools: A Scoping Review

**DOI:** 10.3390/jpm10040223

**Published:** 2020-11-12

**Authors:** Patrick Curtin, Alexandra Conway, Liu Martin, Eugenia Lin, Prakash Jayakumar, Eric Swart

**Affiliations:** 1 Department of Orthopedics, University of Massachusetts Medical Center, 55 N Lake Avenue, Worcester, MA 01655, USA; Patrick.Curtin@umassmemorial.org (P.C.); Alexandra.Conway@umassmed.edu (A.C.); Liu_Martin@branson.org (L.M.); 2 Department of Surgery and Perioperative Care, University of Texas at Austin Dell Medical School, 1601 Trinity Street, Austin, TX 78712, USA; Eugenia.Lin@austin.utexas.edu (E.L.); Prakash.Jayakumar@austin.utexas.edu (P.J.)

**Keywords:** web-based tools, orthopedics, predictive tools

## Abstract

Web-based personalized predictive tools in orthopedic surgery are becoming more widely available. Despite rising numbers of these tools, many orthopedic surgeons may not know what tools are available, how these tools were developed, and how they can be utilized. The aim of this scoping review is to compile and synthesize the profile of existing web-based orthopedic tools. We conducted two separate PubMed searches—one a broad search and the second a more targeted one involving high impact journals—with the aim of comprehensively identifying all existing tools. These articles were then screened for functional tool URLs, methods regarding the tool’s creation, and general inputs and outputs required for the tool to function. We identified 57 articles, which yielded 31 unique web-based tools. These tools involved various orthopedic conditions (e.g., fractures, osteoarthritis, musculoskeletal neoplasias); interventions (e.g., fracture fixation, total joint arthroplasty); outcomes (e.g., mortality, clinical outcomes). This scoping review highlights the availability and utility of a vast array of web-based personalized predictive tools for orthopedic surgeons. Increased awareness and access to these tools may allow for better decision support, surgical planning, post-operative expectation management, and improved shared decision-making.

## 1. Introduction

The ability to provide personalized predictions of clinical outcomes in the field of orthopedics is gaining interest [1,2,3,4,5]. Databases encompassing robust and accurate patient-level data [6,7,8], greater access to patient information via the electronic medical record [9], and the rise of advanced analytical capabilities, such as machine learning [10,11], provide the prospect of great strides in both our understanding of musculoskeletal problems and the outcomes of orthopedic interventions. Unlike simple risk calculations, web-based predictive tools analyze larger amounts of patient data and utilize algorithmic mathematical modeling and prediction analytics using advanced computing.

Despite the technological advances in predictive tools, many challenges exist in practical implementation of these solutions in clinical settings. Firstly, there is no common repository or standardized location to access personal predictive tools. These tools span various subspecialties within the field of orthopedics and other surgical specialties (e.g., capable of providing general risk calculations). As a result, where to find and identify tools appropriate for their clinical needs remains a barrier for orthopedic surgeons in practice. Secondly, once a tool has been identified for use, it can be difficult to discern how the tool was developed (i.e., what data inputs have been used to define the tool’s algorithm) and how it has been assessed for technical feasibility and validated (i.e., the extent to which a tool can be used in a practice setting to fit a given need). Our overarching goal was to perform a scoping review to comprehensively map and organize the knowledge base around web-based personalized predictive tools in orthopedics.

Our primary objective was to map the current range of web-based predictive tools by type of data input, study characteristics and statistical methods used to develop the tool, type of data output, and the function of the tool. Our secondary objective was to qualitatively synthesize this data to generate a set of considerations for disseminating and implementing these tools in routine orthopedic practice. Our findings aim to provide orthopedic surgeons comprehensive insights into the range of available tools, researchers and technologists a premise to develop further innovative solutions in this field, and health systems a framework to integrate these tools to advance facets of orthopedic care.

## 2. Methods

We drafted a protocol a priori using the Preferred Reporting Items for Systematic Reviews and Meta-analysis Protocols (PRISMA-SCR), and members of our research team further refined the protocol through a collaborative process. Our protocol was registered through Open Science Framework on 24 September 2020 [12]. (To be included in this review, articles and studies needed to be in English, and include the uniform resource link (URL) for their web-based tool and/or sufficient information (references, tool name, researcher(s), institution, etc.) to find the tool. The development and validation studies mentioned in these articles needed to describe their patient population, intervention in the form of a personalized web-based predictive tool, methodology and description of their validation and development, outcomes provided by the web-based tool, and type of study. Articles referencing studies for which this information was missing were excluded. Exclusion criteria also included articles without URLs or sufficient information to identify the tool, article links with no access to full-text PDFs, and articles written in languages other than English. Articles about tools with no orthopedic relevance, such as a tool predicting cardiovascular disease risk in patients taking statins, were also excluded from this study. For the purposes of this review, prospective cohort studies, retrospective cohort studies, and meta-analyses were included, while case reports were excluded.

To identify web-based tools that fit the above inclusion criteria, a comprehensive search of the bibliographic electronic database PubMed (NLM) was conducted using specific search terms and with no time-period restriction. Search terms were initially drafted by an experienced orthopedic surgeon familiar with web-based orthopedic tools, and further refined through discussion among the research team. Search results were then screened for the above inclusion criteria and included only if all criteria were met. To ensure the comprehensive capture of any additional web-tools, a second more targeted search was performed, focusing on a set of high-impact orthopedic journals within each subspecialty. Targeted subspecialties are listed in Table 1. The list of high-impact orthopedic journals was created by an experienced orthopedic surgeon and the research team. These journals are listed in Appendix A. Duplicate articles were removed for both searches. After identifying all tools, missing original development and validation articles were found and included for any tools that lacked them in the initial searches. The final search strategy with both primary and secondary searches is recorded in Appendix B.

Screening of the identified articles was performed by all members of the research team. For articles that passed preliminary screening, we downloaded the full-text versions of the studies, mostly as portable document files (PDFs). We subsequently extracted and recorded relevant data using an electronic data collection sheet. For each tool we extracted the following parameters: tool name, functional URL link, user input data, tool outputs, type of study used for tool creation and/or validation, number of patients involved in those studies, and statistical methods used for creation of tool. User inputs were categorized into demographic, clinical, and patient reported data. Clinical input data were defined to include medical, biometric, and radiologic findings. Categorical examples of user input data are listed in Table 2. Tools were then grouped by output category, examples for which are listed in Table 3.

## 3. Results

We identified 358 total records through our search strategy, 57 of which matched our inclusion criteria. From those 57 articles, 31 unique tools were identified, analyzed, and included for this scoping review (Table 4). There was a higher number of articles identified compared to tools because some articles were reviews of multiple tools or re-validation studies of already identified tools. Useable links for each tool are listed in Appendix C. 

**Figure 1:**
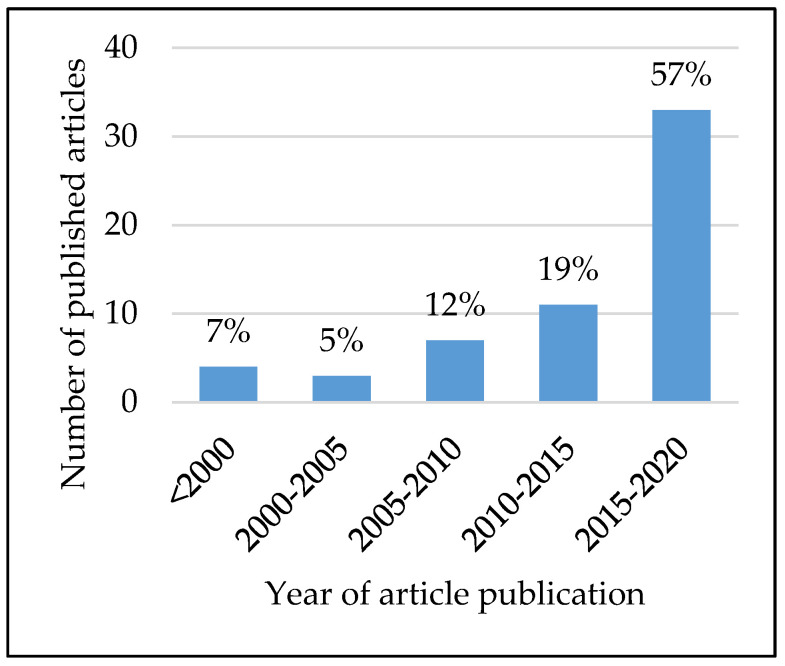
Article publication over time for web-based orthopedic predictive tools.

**Figure 2:**
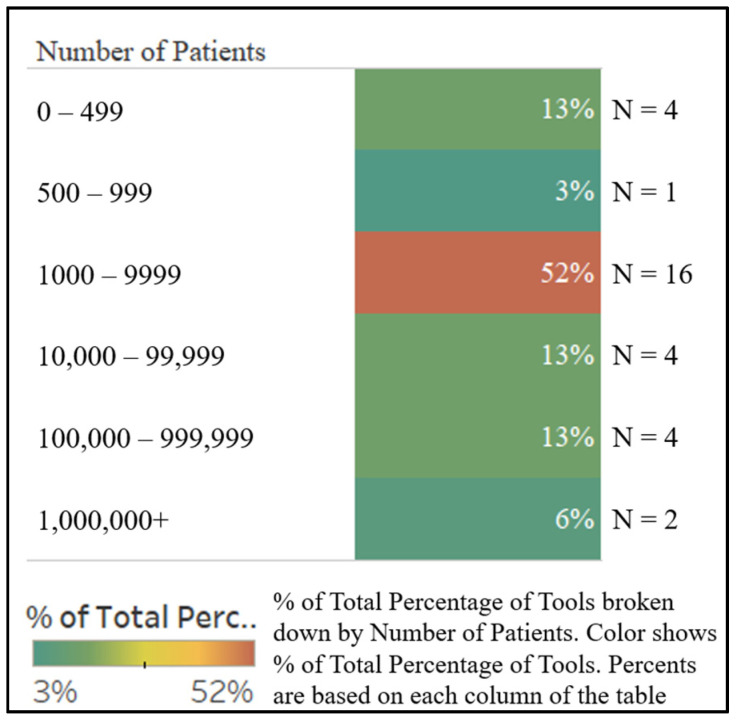
Study size used in tool creation.

**Figure 3:**
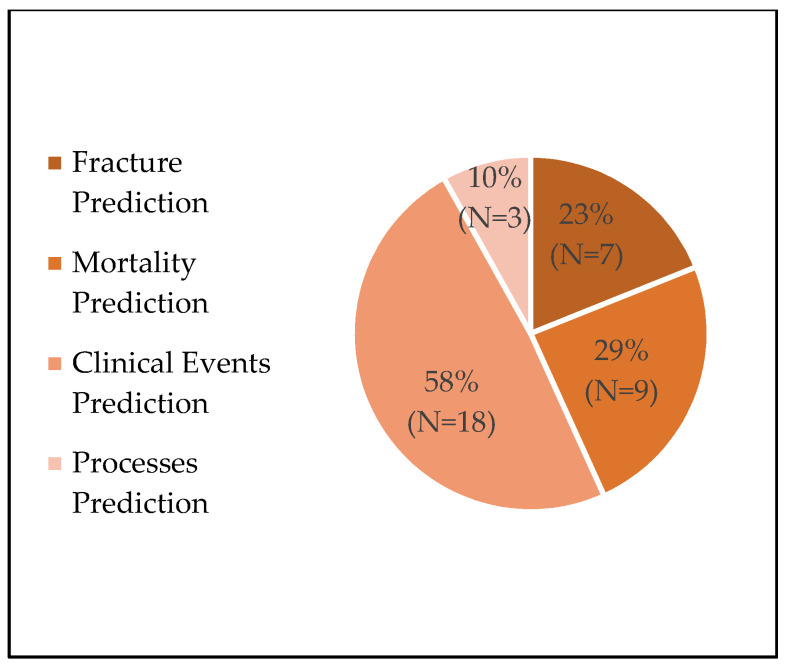
Tool breakdown by output.

**Figure 4:**
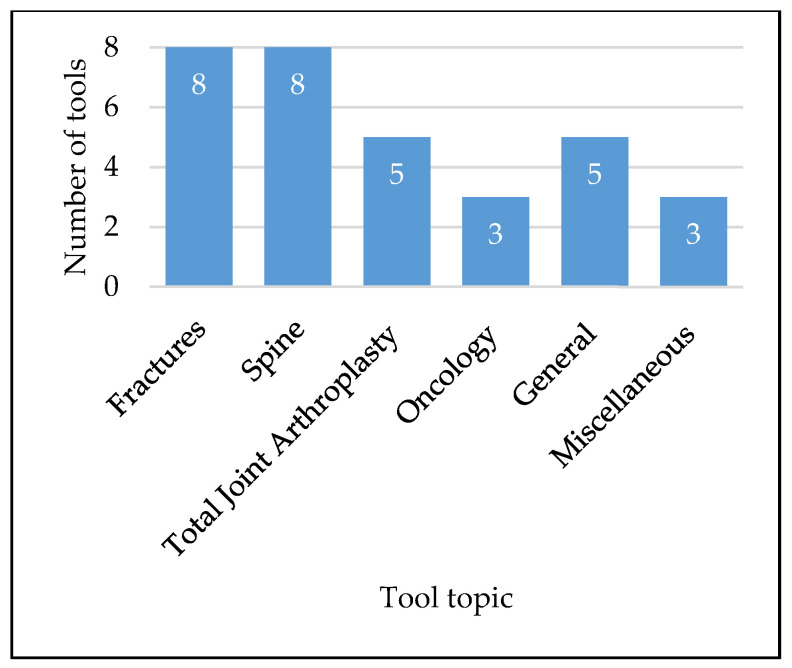
Tool breakdown by orthopedic category.

We found that the frequency of orthopedic web-based tool publications has increased over time, with 7% of articles being published before the year 2000, 17% between 2000 and 2010, and the remaining 76% between 2010 and 2020 (Figure 1). Tool development was more often informed by exclusively retrospective studies (*n* = 17, 55%) than by exclusively prospective studies (*n* = 12, 39%), with only two tools using both retrospective and prospective data sources (*n* = 2, 6%). Additionally, the sizes of the studies used in tool development varied greatly, with a median study size of 2216 patients, an average study size of 241,644 patients, and a range of 257–4,726,046 patients (Figure 2). While nearly all tools were used clinical inputs (*n* = 30, 97%) and demographic inputs (*n* = 29, 94%), a minority used patient-reported inputs (*n* = 12, 39%). Age was the most commonly used demographic input (*n* = 25, 81%), with Sex/Gender as the second-most common (*n* = 16, 52%). Statistical methods used for the creation of tools varied, with most involving logistic regression or multivariate analysis (Table 4). Categorization of the tools by output yielded the following breakdown: fracture prediction (*n* = 7, 23%), mortality prediction (*n* = 9, 29%), clinical event prediction (*n* = 18, 58%), and processes prediction (*n* = 3, 10%) (Figure 3). Finally, we found that most tools could be further categorized into orthopedic categories, such as fractures (*n* = 8, 26%), spine (*n* = 8, 26%), total joint arthroplasty (*n* = 5, 16%), oncology (*n* = 3, 10%), general (*n* = 5, 16%), and miscellaneous (*n* = 3, 10%) (Figure 4).

## 4. Discussion

In this scoping review, we systematically identified 31 web-based tools designed to provide personalized prediction in various of orthopedic settings. Overall, these tools provide orthopedic surgeons with information supporting the outcome prediction of fractures, mortality, other clinical events, such as surgical complications, and miscellaneous clinical processes, such as length of stay. This information applies to settings in different orthopedic subspecialties, in various points of the clinical pathway including pre- and post-operative surgical planning, and for shared decision-making with patients in discussions of elective surgeries.

### 4.1. Limitations

This work should be considered in light of some limitations. Firstly, many identified papers had tools that were inaccessible or had broken URLs. While this may indicate that a given tool was discontinued for technical reasons, it is challenging to ascertain whether there were modifications made for a newer version or alternative use. As tools continue to develop, iterative processes with well-documented updates and modifications will need to be utilized to better implement these tools in clinical practice. Secondly, papers presented varying levels of detail regarding the tool development process which limited standardization of specific parameters. Future work should aim to increase transparency in the tool development and validation processes so that tool comparisons are uniform and systematic. Thirdly, only one database was used to search for these tools. While this raises the possibility of missed tools, the two searches conducted for this study were broad and anticipated the capture of the vast majority of these web-based orthopedic solutions. The selected electronic database (PubMed) is also the most relevant to clinical orthopedic practice. Finally, there may be more tools in development that are currently being validated at various institutions and remain unpublished at the time of this review. While this is largely unavoidable in a fast-paced field, further work may target the grey literature and other types of electronic databases or search engines to capture such tools.

It is difficult to quantify the current use of web-based orthopedic predictive tools in practice. This scoping review demonstrates a rapid increase in the frequency of tool-related publications over the last two decades, perhaps reflecting growing interest in web-based orthopedic predictive tools. Although specialized commercial software packages may overshadow the use of web-based tools in areas such as pre-operative planning, web-based tools offer additional and unique functions, such as patient and surgical outcome predictions. In this way, web-based tools serve as potential complements to already established software packages.

Identified tools consistently rely on more established statistical methods for their development, such as multivariate analysis and logistic regression. There were only two tools that demonstrate the use of machine-learning algorithms in their development, both of which were released within the past 5 years. Artificial intelligence and machine learning have the ability to process large amounts of data in different forms, including actively and passively generated data from patients. It is likely that, as this technology evolves, future web-based medical tools will make increasing use of advanced predictive analytics and provide greater opportunities for more personalized patient care.

### 4.2. Future Work

The profile of tools identified in this study indicate two major areas for potential improvement. First, fewer than half of identified tools utilize patient-reported inputs. This may be related to the reliability of obtaining information from patients, as these data require active procurement in prospective cohorts or may not be consistently documented for retrospective cohorts. Despite these barriers, patient reported measures are important components of patient outcomes and ensure physician focus on subjective metrics that patients care about, such as perceived pain. As patient-reported outcome measures are increasingly used in both the field of orthopedics and across medical specialties, greater incorporation of patient-reported data into these tools presents an area of potential improvement. Second, tools identified in this study fall into just a few orthopedic categories, such as fractures, total joint arthroplasty, spine, and oncology. These categories may reflect a lack of tools for other orthopedic subspecialties, such as orthopedic sports injuries. This lack may be related to the heterogeneity of injuries in such fields or insufficient access to large existing patient databases for tool development. Continued expansion in the development of tools across orthopedic subspecialties would afford a wider breadth of resources to orthopedic surgeons to better clinical outcomes for patients and should be a focus of improvement.

## 5. Conclusions

The increasing number of web-based orthopedic tools is an opportunity for orthopedic surgeons to better predict outcomes and increase understanding of expectations with patients. The aim of this scoping review was to identify the current list of web-based orthopedic tools, as well as clearly outline their utility and validation. We provide orthopedic surgeons a repository of current and publicly available web-based tools which includes all the necessary information to determine what tools may apply to their practice. Areas for continued development of web-based tools are vast, with opportunities in both tool design and application.

## Figures and Tables

**Table 1:** Targeted Orthopedic Subspecialties Used in Secondary Search.

Joint Replacement
Research
Trauma
Sports
Hand and Upper Extremity
Shoulder and Elbow
Foot and Ankle
Spine
Pediatric

**Table 2:** Types of User Input Data.

Category	Examples
Demographic	Age, Sex, Body Mass Index (BMI)
Clinical	Medical History, Injury Characteristics, Procedure Characteristics
Patient Reported	Social Information (e.g., housing, sexual activity, recreational activities, etc.), Smoking Status, Current Alcohol Use, Recent Fall History

**Table 3:** Types of Tool Output Data.

Category	Examples
Fracture Prediction	Loss of Position, Risk of Fracture
Mortality Prediction	Survival Rates
Clinical Events Prediction	Surgical Complications, Post-Operative Pain, Readmission Rates, Treatment Options
Processes Prediction	Length of Stay, Discharge Disposition

Data were checked by multiple team members (P.C./L.M./A.C.) to ensure accurate collection. We defined the data extraction elements by consensus and managed any inconsistencies and disagreements from data screening and extraction via consensus discussions and rounds of voting. There were no disagreements that were unable to be resolved, so no extra party was needed to act as a tiebreaker.

**Table 4:** Tools Categorized by Output.

Tool	Journal and (Year) of Publication [Ref]	Tool Development Study Type and Size	Demographic Input	Clinical Input	Patient Reported Input	Tool Output	Statistical Methods Used in Tool Development
Edinburgh Wrist Calculator	*Journal of Orthopaedic Trauma* (2018) [13,14], *The Journal of Bone and Joint Surgery* (2006) [15]	Prospective Cohort 4000 distal radius fractures	Age	Ulnar Variance, Dorsal Comminution Present, Physical Dependence		Fracture prediction: Loss of position	Univariate and Multiple Logistic Regression
CAROC (Canadian Risk for Osteoporosis Calculator)	*Journal of Clinical Densitometry* (2017) [16], (2010) [17], (2007) [18]	Retrospective Cohort 39,603 patients	Age, Sex	Femoral Neck T-Score, Fragility Fracture after age 40, Recent prolonged glucocorticoid use		Fracture prediction: Risk categorization	Kaplan–Maier Method
FRAX	*Journal of Clinical Densitometry* (2017) [19], *Turkish Journal of Urology* (2019) [20]	Systematic Review 290,000 patients	Age, Sex, Height, Weight	Previous Fracture, Glucocorticoids, Rheumatoid arthritis, Secondary Osteoporosis, Femoral neck BMD	Smoking status, Alcohol use, Parental history of hip fracture	Fracture prediction: 10-year risk	Multiple Logistic Regression
FRS (Fracture Risk Scale)	*BioMed Central (BMC) Geriatrics* (2018) [21], *British Medical Journal (BMJ) Open* (2017) [22]	Retrospective Cohort 29,848 patients	Age, BMI	Wandering frequency, Walking in corridor, Transfer status, Cognitive performance scale, hip fracture history	Fall history	Fracture prediction: 1-year hip fracture risk	Decision Tree and Logistic Regression
Garvan	*Journal of Clinical Densitometry* (2017) [19], *Osteoporosis International* (2008) [23]	Prospective Cohort 2216 patients	Age, Sex	Fractures and fall history, T-scores, actual BMD		Fracture prediction: 5- and 10-year risk	Cox’s Proportional Hazards Analysis
QFracture	*Journal of Clinical Densitometry* (2017) [16,19], *The British Medical Journal* (Clinical research ed.) (2012) [24]	Prospective Cohort 4,726,046 patients	Age, Sex, BMI, Ethnicity	Diabetes, osteoporotic fracture history, Dementia, Cancer, Asthma or COPD, Cardiovascular disease, liver disease, kidney disease, Parkinson’s, Rheumatoid arthritis, SLE, GI malabsorption, Endocrine problems, Epilepsy, Hormone Therapy, use of anticonvulsants/ antidepressants/corticosteroids/estrogen	Smoking status, Alcohol use, Fall history, Parental history of hip fracture/osteoporosis, Residence	Fracture prediction: 10-year risk	Multivariate Final Cox Regression
NHFS (Nottingham Hip Fracture Score)	*British Journal of Anaesthesia* (2008) [25], *The Bone & Joint Journal* (2015) [26], *Injury* (2015) [27]	Prospective Cohort 4967 patients	Age, Sex	AMTS, Hb on admission, Comorbidities, Active malignancy history	Residence	Fracture prediction: NHFS Score Mortality prediction	Forward Univariate and Multivariate Logistic Regression
ACS NSQIP Surgical Risk Calculator	*Journal of Neurosurgery. Spine* (2017) [28], *The Journal of Arthroplasty* (2018) [29], *Spine* (2020) [30], *Clinical Orthopaedics and Related Research* (2016) [31], *The Journal of Arthroplasty* (2015) [32], *Journal of the American College of Surgeons* (2013) [33]	Retrospective Cohort 1,414,006 patients	Age, Sex, Height, Weight	Procedure, Functional Status, Emergency Case, ASA Class, Steroid use, Ascites, Systemic Sepsis, Ventilator Dependent, Disseminated Cancer, Diabetes, Hypertension, CHF, Dyspnea, History of Severe COPD, Dialysis, Acute Renal Failure	Smoking status	Mortality prediction Clinical events prediction: Adverse events Processes prediction: Length of stay	Random Intercept and Fixed Slope Hierarchical Models
E-PASS (Estimation of Physiologic Ability and Surgical Stress) Surgery Risk Calculator	*Journal of Bone and Mineral Research* [34], *Injury* (2015) [27], *Surgery Today* (1999) [35], *Surgery* (2004) [36]	Retrospective Cohort 3981 patients	Age, Weight	Cardiac arrhythmia, Pulmonary vital capacity, FEV1, Diabetes, Blood loss, OR time, Extent of skin incision at surgery, Heart failure, ECOG performance status, ASA class		Clinical event prediction: Preoperative risk score, Surgical stress score, Comprehensive risk score	Multiple Logistic Regression
STTGMA (Score for Trauma Triage in Geriatric and Middle Aged Patients)	*The Journal of the American Academy of Orthopaedic Surgeons* (2020) [37], *Bulletin of the Hospital for Joint Disease* (2016) [38]	Retrospective Cohort 138,096 patients	Age	Injury mechanism, Glasgow Coma Scale (GCS), Abbreviated Injury Scale (AIS), Head/Neck, Chest, Extremity, Charlson Comorbidity Index (CCI)		Mortality prediction: STIGMA Score	Logistic Regression Analysis
CCI (Charlson Comorbidity Index)	*Journal of Neurosurgery. Spine* (2017) [28], *Clinical Orthopaedics and Related Research* (2014) [39], *Injury* (2015) [27], *Journal of Orthopaedic Research* (2020) [40], Journal of Chronic Diseases (1987) [41]	Prospective Cohort 1244 patients	Age	Myocardial infraction, CHF, Peripheral vascular disease, CVA or TIA, Dementia, COPD, Connective tissue disease, Peptic ulcer disease, Liver disease, Diabetes Mellitus, Hemiplegia, Moderate to severe CKD, Solid tumor, Leukemia, Lymphoma, AIDS		Mortality prediction: CCI, 10-year survival	Kaplan–Maier Method
P-POSSUM	*The British Journal of Surgery* (1991) [42], (1998) [43], *Injury* (2015) [27]	Prospective Cohort 1440 patients	Age	Cardiac status, Respiratory status, ECG, Systolic BP, Pulse, Hemoglobin, WBC, Urea, Sodium, Potassium, GCS, Operation type, Number of procedures, Operative Blood Loss, Peritoneal Contamination, Malignancy Status, CEPOD		Mortality prediction (for esophagogastric surgery) Clinical event prediction: Physiology score, Operative severity score, Morbidity	Multiple Logistic Regression
LACE+	*Journal of Orthopaedic Research* (2020) [40], *Canadian Medical Association Journal* (2010) [44], *Open Medicine* (2012) [45]	Prospective Cohort 500,000 patients		Length of stay, Acuity of admission, Comorbidities, Emergency department visits		Mortality prediction: LACE+ score Clinical event prediction: Readmission risk	Split-Sample Design, Fractional Polynomial Functions, Multiple Logistic Regression
Chondrosarcoma Five-Year Survival Machine Learning Algorithm	*Clinical Orthopaedics and Related Research* (2018) [46]	Retrospective Cohort 1554 patients	Age, Sex	Histology, Size, Extension, Grade, Location		Mortality prediction: 5-year survival	Nonparametric Missforest Method, Boosted Decision Tree, Support Vector Machine, Bayes Point Machine, Neural Network Models, 10-Fold Crossvalidation
Extremity Metastatic Disease Survival Prediction Machine Learning Algorithm	*Clinical Orthopaedics and Related Research* (2020) [47]	Retrospective Cohort 1090 patients	Age	Primary Tumor Histology, Visceral Metastasis, Brain Metastasis, Previous Systemic Therapy, Hemoglobin, Platelet, Absolute Lymphocyte, Absolute Neutrophil, Creatinine, White Blood Cell, Albumin, Alkaline Phosphatase, Sodium, Calcium		Mortality prediction: 90-day and 1-year survival	Missforest Methods, Random Forest Algorithms, Stochastic Gradient Boosting, Random Forest, Support Vector Machine, Neural Network, and Penalized Logistic Regression
PathFX	*Clinical Orthopaedics and Related Research* (2017) [48], *BioMed Central (BMC) Cancer* (2015) [49]	Retrospective Cohort 1291 patients	Age, Sex	Oncologic Diagnosis, Pathologic Fracture, ECOG Performance Status, Hemoglobin concentration, Absolute lymphocyte count, Skeletal Metastases, Organ Metastases, Lymph Node Metastases, Physician’s Estimate of Survival, Skeletal Region		Mortality prediction: Survival likelihood after treatment at multiple time intervals Clinical event prediction: Potential treatments	Bayesian Belief Networks
Incidental Durotomy Calculator	*Journal of Neurosurgery. Spine* (2020) [50]	Retrospective Review 1279 patients	Age	Revision Procedure, Procedure start after 4 pm, Surgery duration		Clinical event prediction: Incidental durotomy likelihood	Natural language processing (NLP), Multiple Logistic Regression, Bootstrapping
Spinal RAT (Risk Assessment Tool)	*Journal of Neurosurgery. Spine* (2017) [28]	Prospective and Retrospective Cohort 279,391 patients	Age, Sex	Spinal Area, Pre-op diagnosis, Use of BMP, Fusion, Surgery level, Instrumentation, Pulmonary dysfunction, Neurologic dysfunction, Hypercholesterolemia, Hypertension, Cardiac dysfunction, Diabetes mellitus, Systemic malignancy, Gastroesophageal dysfunction, Psychiatric disorder, Substance abuse	Smoking status	Clinical event prediction: Surgical complications risk (%), risk classification (low, medium, high)	Logistic Regression with Main Effects, 2 and 3 Factor Interactions
SpineSage	*The Spine Journal* (2014) [51]	Prospective Cohort 1532 patients	Age, Gender, BMI	Primary Diagnosis, Level of Surgery, Surgical Approach, Cerebrovascular Disease, Chronic Obstructive Pulmonary Disease, Asthma, Hypertension, Rheumatoid Arthritis, Renal Conditions, Pre-existing Neoplasm, Syncope or Seizure, Anemia, Bleeding disorder, Diabetes, CHF, Revision surgery, Previous spinal surgery, Previous cardiac complications		Clinical event prediction: Complications risk	Multivariate Analysis
Back Treatment Outcomes Calculator	*Spine* (2002) [52], (2018) [53]	Prospective Cohort 289 patients	Age, Sex, Height, Weight	Condition, Symptoms, Episode, Hypertension, Physical therapy, Depression	Smoking status, Activities, Work status, Worker’s compensation, Education, Expectation, Sleep, Sex life	Clinical event prediction: Treatment risks, Outcomes with or without surgery (physical functioning, pain, sleep, sex life, satisfaction with symptoms)	Multivariate Analysis
OaraScore (Outpatient Arthroplasty Risk Assessment Score)	*The Journal of Arthroplasty* (2018) [54], (2017) [55]	Retrospective Review 1120 patients	BMI	Chronic narcotic use, Chronic pain control difficulty, Chronic benzodiazepine use, Severe deconditioning; Additional inputs by topic	Lack of home support	Clinical event prediction: Outpatient surgery risk, Assessment score	Multivariate Analysis
Arthroplasty Size Predictor	*The Journal of Arthroplasty* (2019) [56], (2017) [57]	Retrospective Cohort 3491 primary TKAs	Sex, Height, Weight	Manufacturer, Model		Clinical prediction: Predicted sizes	Multivariate Linear Regression
90-Day Readmissions Risk Calculator	*The Journal of Bone and Joint Surgery* (2019) [58]	Retrospective Cohort 10,155 THAs and TKAs	Age	Joint, ASA, Duration of Surgery, Hemoglobin Level Postoperative, Cardiac Arrhythmia, CHF, Chronic Pulmonary Disease, Diabetes Complicated, Hypertension, Lymphoma, Neurologic Disease, Peripheral Vascular Disease, Pulmonary Circulation Disease, Renal Failure, Depression, Substance abuse	Smoking Status, Alcohol use	Clinical event prediction: 90-day readmission risk	Multiple Logistic Regression
QUALITOUCH Outcome Calculator	*Journal of Telemedicine and Telecare* (2014) [59]	Prospective Cohort 483 patients	Age, Sex, Height, Weight	Type of surgery, Hip replacement, Knee replacement, Spine surgery, Hypertension, Heart disease, Stroke, Depression, Diabetes, Cancer, Lung/Kidney/GI disease, Anemia, Substance abuse	Pain during activities, Difficulty in activities/movements, Chronic pain, Back pain	Clinical event prediction: Current pain level, 3-month post-op predicted pain level	Multiple Regression
STaRT Back Tool	*Arthritis Care & Research* (2008) [60], *The Journal of Arthroplasty* (2019) [61], *Spine* (2002) [53]	Prospective Cohort and Retrospective Review 1641 patients		Symptoms	Activity, Pain level, Mental state	Clinical event prediction: Chronic pain risk level	Forward Stepwise Binary Logistic Regression Analysis
Estimated Blood Loss Calculator	*The Spine Journal* (2020) [62]	Retrospective Cohort 1281 patients	BMI	Pedicle Screws (T11-S1), Pelvic Screws, Laminectomy, Laminectomy Levels, Discectomies, ALIF Interbody Fusions, XLIF/OLIF Interbody Fusions, TLIF/PLIF Interbody Fusions, Schwab Osteotomies, TXA Use, Surgery duration		Clinical event prediction: Blood loss (mL)	Univariate Linear Regressions, Multivariate Analysis
ShockNurd	*Clinical Orthopaedics and Related Research* (2016) [63]	Retrospective Review 382 patients	Sex	Tibial nail >4 weeks ago, Percentage Cortical Contact, Open Fracture, Compartment Syndrome, Soft Tissue Flap Required, Chronic Condition (HIV/HEP C/Diabetes), ASA Classification, Low Energy Injury, Spiral Fracture Pattern		Clinical event prediction: NURD Score, Non-Union Percentage, Confidence Range	Bivariate and Multivariate Regression, Stepwise Modeling
Neuro Risk Opioid Use Calculator	*Spine* (2018) [64]	Retrospective Cohort 26,553 patients	Age, Gender	Cervical or Lumbar Spine, Operation type, Diabetes, Depression/Anxiety, Osteoporosis, Fibromyalgia, Morbid Obesity, Lower Back Pain, Motor Deficits (plegia), Bowel/Bladder dysfunction, Substance abuse, Preoperative Opioid User (3 Months Prior to Surgery),		Clinical event prediction: Narcotics use at 12-month postop	Multiple Logistic Regression
Opioid Calculator for Hand Surgery	*The Journal of Hand Surgery* (2019) [65]	Prospective Cohort 526 patients	Age	Can take Naproxen post-op, Can take Acetaminophen post-op, Currently taking Narcotics, Planned use of regional anesthesia, Procedure involves bone/ligament, Anticipated Surgical Time		Clinical event prediction: Number of pills to prescribe	Bivariate Analysis and Multiple Logistic Regressions
Discharge to Rehabilitation and LOS Calculator	*The Spine Journal* (2020) [66]	Retrospective Cohort 257 patients	Age, BMI, Insurance	Diabetic, Type of spine surgery, Procedure time, Elective vs. Emergent		Processes prediction: Risk of discharge to rehab, Length of stay	Univariable And Multivariable Analyses
RAPT (Risk Assessment and Prediction Tool)	*The Journal of Arthroplasty* (2019) [67], (2020) [68], (2019) [61], *Clinical Orthopaedics and Related Research* (2015) [69]	Prospective Cohort 3213 patients	Age, Sex		Functional Abilities, Social Support	Processes prediction: Discharge requirements, Length of stay	Binary Logistic Regression

Abbreviations: ALIF—Anterior Lumbar Interbody Fusion, AMTS—Abbreviated Mental Test Score, BMD—Bone Mineral Density, BMP—Bone Morphogenetic Protein, CEPOD—Confidential Enquiry into Peri-Operative Deaths, CHF—Congestive Heart Failure, CKD—Chronic Kidney Disease, COPD—Chronic Obstructive Pulmonary Disease, CVA—Cerebrovascular Accident, ECG—Echocardiogram, ECOG—Eastern Cooperative Oncology Group, FEV1—Forced Expiratory Volume, GCS—Glasgow Coma Scale, PLIF—Posterior Lumbar Interbody Fusion, SLE—Systemic Lupus Erythematosus, TIA—Transient Ischemic Attacks, TXA—Tranexamic Acid, XLIF—Extreme Lateral Interbody Fusion, OLIF—Oblique Lumbar Interbody Fusion, TLIF—Transforaminal Lumbar Interbody Fusion.

**Figure A1:**
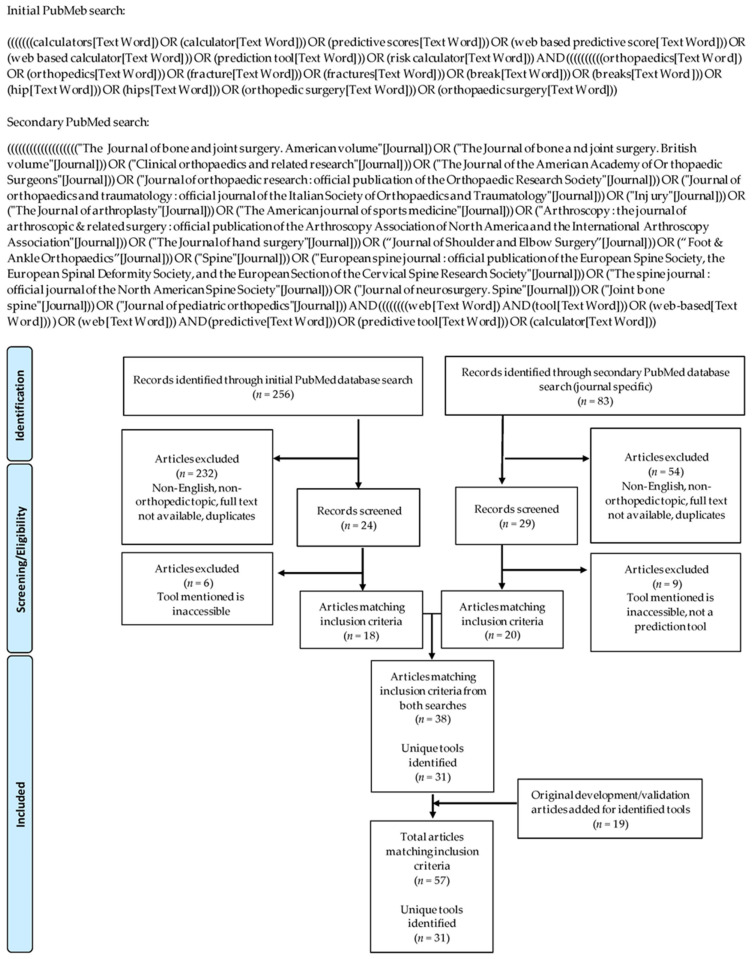
Search Strategy and Identification of Sources.

## Appendix A

**Table A1:** High Impact Orthopedic Journals Used in Secondary Search.

*The Journal of Bone and Joint Surgery, American Volume*
*The Bone and Joint Journal*
*Clinical Orthopaedics and Related Research*
*The Journal of the American Academy of Orthopaedic Surgeons*
*Journal of Orthopaedic Research: Official Publication of the Orthopaedic Research Society*
*Journal of Orthopaedics and Traumatology: Official Journal of the Italian Society of Orthopaedics and Traumatology*
*Injury*
*The Journal of Arthroplasty*
*The American Journal of Sports Medicine*
*Arthroscopy: The Journal of Arthroscopy & Related Surgery: Official Publication of the Arthroscopy Association of North America and the International Arthroscopy Association*
*The Journal of Hand Surgery*
*Journal of Shoulder and Elbow Surgery*
*Foot & Ankle Orthopaedics*
*Spine*
*European Spine Journal: Official Publication of the European Spine Society, the European Spinal Deformity Society, and the European Section of the Cervical Spine Research Society*
*The Spine Journal: Official Journal of the North American Spine Society*
*Journal of Neurosurgery. Spine*
*Joint Bone Spine*
*Journal of Pediatric Orthopedics*

## Appendix C

**Table A2:** Tool URLs.

Tool	URL	Additional Notes
90-Day Readmissions Risk Calculator	https://dukeriskcalculators.shinyapps.io/Readmissions/ (accessed on 26 October 2020)	
ACS NSQIP Surgical Risk Calculator	https://riskcalculator.facs.org/RiskCalculator/ (accessed on 26 October 2020)	Option to email or download report as PDF
Arthroplasty Size Predictor	https://apps.apple.com/us/app/arthroplasty-size-predictor/id1234761373 (accessed on 26 October 2020)	iOS application
Back Treatment Outcomes Calculator	http://spinesurgerycalc.dartmouth.edu/calc/ (accessed on 26 October 2020)	
CAROC (Canadian Risk for Osteoporosis Calculator)	https://osteoporosis.ca/health-care-professionals/tools/caroc/ (accessed on 26 October 2020)	
CCI (Charlson Comorbidity Index)	https://www.mdcalc.com/charlson-comorbidity-index-cci (accessed on 26 October 2020)	Free registration required; option to copy results to clipboard as text
Chondrosarcoma Five-Year Survival Machine Learning Algorithm	https://sorg-apps.shinyapps.io/chondrosarcoma/ (accessed on 26 October 2020)	
Discharge to Rehabilitation and LOS Calculator	https://jhuspine1.shinyapps.io/RehabLOS/ (accessed on 26 October 2020)	
Edinburgh Wrist Calculator	https://www.trauma.co.uk/wristcalc (accessed on 26 October 2020)	
E-PASS (Estimation of Physiologic Ability and Surgical Stress) Surgery Risk Calculator	https://www.medicalalgorithms.com/surgery-risk-calculator-estimation-of-physiologic-ability-surgical-stress (accessed on 26 October 2020)	
Estimated Blood Loss Calculator	https://jhuspine2.shinyapps.io/Estimated_Blood_Loss/ (accessed on 26 October 2020)	
Extremity Metastatic Disease Survival Prediction Machine Learning Algorithm	https://sorg-apps.shinyapps.io/extremitymetssurvival/ (accessed on 26 October 2020)	
FRAX	https://www.sheffield.ac.uk/FRAX/ (accessed on 26 October 2020)	Desktop application; payment required
FRS (Fracture Risk Scale)	https://www.fco.ngo/blog/fracture-risk-scale-frs-tool-assessing-fracture-risk-long-term-care-ltc (accessed on 26 October 2020)	
Garvan	https://www.garvan.org.au/bone-fracture-risk (accessed on 26 October 2020)	Option to print results
Incidental Durotomy Calculator	https://jhuspine3.shinyapps.io/Incidental_Durotomy_Calculator/ (accessed on 26 October 2020)	
LACE+	https://www.besler.com/lace-risk-score/ (accessed on 26 October 2020)	
Neuro Risk Opioid Use Calculator	http://neuro-risk.com/opiod-use/ (accessed on 26 October 2020)	
NHFS (Nottingham Hip Fracture Score)	http://www.riskprediction.org.uk/index-nhfs.php (accessed on 26 October 2020)	
OaraScore (Outpatient Arthroplasty Risk Assessment Score)	https://www.djoglobal.com/our-brands/djo-surgical/oara-score (accessed on 26 October 2020)	Paid subscription required; option to print results
Opioid Calculator for Hand Surgery	https://jscalc.io/calc/9hH05AdFRt4iV6YD (accessed on 26 October 2020)	
PathFX	https://www.pathfx.org/ (accessed on 26 October 2020)	Free registration required
P-POSSUM	http://www.riskprediction.org.uk/index-op.php (accessed on 26 October 2020)	
QFracture	https://qfracture.org/ (accessed on 26 October 2020)	
QUALITOUCH Outcome Calculator	https://outcomecalculator.org/en/ (accessed on 26 October 2020)	
RAPT (Risk Assessment and Prediction Tool)	https://www.lifespan.org/centers-services/total-joint-center-lifespan-orthopedics-institute/take-rapt-assessment (accessed on 26 October 2020)	
ShockNurd	http://shocknurd.org/ (accessed on 26 October 2020)	Link currently inactive
Spinal RAT (Risk Assessment Tool)	https://apps.apple.com/us/app/risk-assessment-tool-for-spine-surgery-procedures/id1087663216 (accessed on 26 October 2020)	iOS application
SpineSage	https://depts.washington.edu/spinersk/ (accessed on 26 October 2020)	
STaRT Back Tool	https://startback.hfac.keele.ac.uk/training/resources/startback-online/ (accessed on 26 October 2020)	
STTGMA (Score for Trauma Triage in Geriatric and Middle Aged Patients)	https://sttgma.wordpress.com/ (accessed on 26 October 2020)	Option to download Excel formula sheets
